# Evaluating the effect of γ‐oryzanol on MASLD pathology using a medaka fish model

**DOI:** 10.1002/2211-5463.70301

**Published:** 2026-06-30

**Authors:** Yukako Ito, Haduki Asano, Ayano Ueki, Joe Sakamoto, Yasuhiro Kamei, Hayato Tokumoto, Ayaka Yazawa, Shigeki Kamitani

**Affiliations:** ^1^ Department of Clinical Nutrition Graduate School of Comprehensive Rehabilitation, Osaka Prefecture University Osaka Japan; ^2^ Laboratory for Biothermology National Institute for Basic Biology Okazaki Aichi Japan; ^3^ Optics and Imaging Facility National Institute for Basic Biology Okazaki Aichi Japan; ^4^ Department of Basic Biology School of Life Science, The Graduate University for Advanced Studies (SOKENDAI) Okazaki Aichi Japan; ^5^ Department of Biology Graduate School of Science, Osaka Metropolitan University Osaka Japan; ^6^ Department of Nutrition Graduate School of Human Life and Ecology, Osaka Metropolitan University Osaka Japan; ^7^ Present address: Division of Biophotonics National Institute for Physiological Sciences Okazaki Japan; ^8^ Present address: Biophotonics Research Group Exploratory Research Center on Life and Living Systems Okazaki Japan

**Keywords:** gut microbiota, inflammation, MASH, MASLD, medaka, *γ*‐oryzanol

## Abstract

Metabolic dysfunction‐associated steatotic liver disease (MASLD) encompasses liver conditions not caused by alcohol, including metabolic dysfunction‐associated steatohepatitis (MASH), which involves inflammation and fibrosis, with rising global prevalence. While research to explore MASLD/MASH prevention or cure has largely relied on rodent models, ethical concerns regarding animal welfare have prompted the exploration of alternatives. This study examines the application of a recently developed medaka fish MASH/MASLD model to assess the impact of food components on liver pathology. Medaka were divided into groups and fed for 12 weeks with either a normal diet (ND), a high‐fat diet (HFD), or HFD supplemented with *γ*‐oryzanol (Ory). Liver samples were analyzed morphologically, histologically, and biochemically using GC/MS and real‐time PCR, with gut microbiota composition also assessed. We show that *γ*‐oryzanol supplementation resulted in smaller, fewer lipid droplets in the liver compared with the HFD group, while GC/MS analysis showed a decreasing trend in total and individual fatty acid content. Additionally, gut microbiota diversity improved in the Ory group. These findings align with previous rodent studies, suggesting that *γ*‐oryzanol may suppress hepatic fat accumulation and inflammation. The study demonstrates the potential of *γ*‐oryzanol as a functional food ingredient for preventing MASLD. Furthermore, this study supports the use of medaka as a cost‐effective and ethical alternative to rodent models in food science research.

## Graphical abstract

This study explores a liver disease called MASLD, which is increasing worldwide and can lead to serious damage. Researchers used medaka fish instead of rodents to test a food compound, *γ*‐oryzanol. Fish fed this compound had less liver fat and healthier gut bacteria. The results suggest *γ*‐oryzanol may help prevent liver disease safely and ethically in the future.

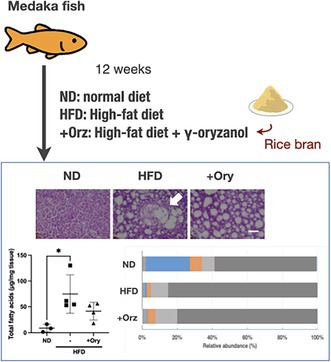

**Abbreviations**
GC/MS: gas chromatography/mass spectrometry
HFD: high‐fat diet
LEfSe: linear discriminant analysis effect size
MASH: metabolic dysfunction‐associated steatohepatitis
MASLD: metabolic dysfunction‐associated steatotic liver disease
ND: normal diet
PCR: polymerase chain reaction

Metabolic dysfunction‐associated steatotic liver disease (MASLD) is a general term for fatty liver conditions that occur without a history of excessive alcohol consumption, and it is commonly known to be closely associated with obesity and diabetes [1]. In recent years, the number of MASLD patients worldwide has been increasing, with approximately 30% of the population in the United States estimated to be affected [2]. MASLD is broadly classified into steatotic liver disease (SLD) and metabolic dysfunction‐associated steatohepatitis (MASH), which involves inflammation, ballooning degeneration of hepatocytes, and fibrosis. MASH is a progressive liver disease that can lead to cirrhosis and eventually liver cancer. A large‐scale survey in Japan reported a MASH prevalence of approximately 3%, suggesting that over 2 million patients have MASH [3].

The ‘two hits hypothesis’ has been proposed to explain the pathogenesis of MASH [4]. The first hit involves fat accumulation in the liver due to a high‐fat diet (HFD) and lack of exercise. The second hit includes oxidative stress, cytokine release, and insulin resistance, which further damage hepatocytes and promote inflammation and fibrosis, leading to MASH. However, this hypothesis is considered insufficient to explain all cases of MASH, and the ‘multiple hits hypothesis’ has become the more widely accepted model [5]. This theory suggests that multiple factors, either simultaneously or sequentially, trigger liver inflammation, promoting the progression of MASH. These factors include activation of fatty acid synthesis pathways in the liver, increased inflammatory cytokines from fat tissue accumulated due to high‐fat, high‐carbohydrate diets, and disturbances in the gut microbiota (dysbiosis) caused by diet and lifestyle, all of which have been reported to be associated with MASH [6].

Due to the complexity and many unknowns in MASH pathogenesis, research continues to evolve. Traditionally, MASH research has relied on rodent models; however, these models often fail to reflect the clinical conditions observed in human patients accurately and are not suitable for large‐scale screening [7]. Moreover, ethical concerns about animal welfare have made it increasingly difficult to use mammals in food‐related research worldwide. For example, in 2019, the Wellcome Sanger Institute, one of the world's leading genomics centers, decided to close its animal research facility after 13 years of operation [8].

Experiments using mammals also require specialized facilities and are costly. In contrast, small fish species such as medaka fish (*Oryzias latipes*) and zebrafish are physiologically and anatomically comparable to higher organisms. They are amenable to genetic manipulation, making them suitable alternative models for human disease research [9, 10]. Medaka fish offer several advantages: (1) They are omnivorous and possess similar carbohydrate and lipid metabolism functions to mammals. (2) Screening can be efficiently conducted by dissolving substances in the water or adding them to the feed. (3) They reproduce rapidly, mature quickly, and are small, reducing research costs. Given these advantages, a MASH model medaka was recently developed by Matsumoto *et al*. [11, 12]. This model involves feeding medaka a HFD for 12 weeks, leading to liver enlargement, widespread hepatic fat deposition, and inflammatory infiltration of hepatocytes. Partial liver fibrosis has also been reported [11, 12]. Unlike traditional mammalian models, the medaka MASH model develops the disease without genetic manipulation, simply through excessive lipid intake, as in human MASH patients. Therefore, it is considered a model that more closely resembles human pathology.

However, due to medaka's small size, the amount of tissue that can be collected from a single fish is limited, requiring advanced dissection and experimental techniques. Additionally, medaka are delicate and may die during experiments. Considering both the advantages and limitations, if the effects of food components on MASH can be reliably evaluated in medaka, it would be possible to conduct large‐scale screening at lower cost and in smaller spaces than in mammalian models. These advantages could significantly contribute to food science and nutritional research as a novel model for disease studies.

MASH is a lifestyle‐related disease closely associated with diet, yet no established treatment currently exists, and research into therapeutic approaches is ongoing [13]. While previous studies have investigated therapeutic drugs using the MASH model medaka [13, 14], research examining the effects of food components using this model remains scarce. Therefore, this study aims to evaluate the utility of the MASH model medaka in food science research and to investigate the effects of food components on MASH.

This study focused on *γ*‐oryzanol, a food component that influences the pathogenesis of typical liver diseases, and conducted experiments to investigate its effects. *γ*‐Oryzanol is a component primarily found in rice bran. While opportunities for natural intake are limited, it is known to possess anti‐inflammatory [15] and antioxidant [16] effects. Furthermore, mouse studies have reported that it mitigates dependence on animal fats by suppressing endoplasmic reticulum stress, raising expectations for its impact on obesity and diabetes [17]. Another prior study found that feeding rats a diet containing 0.16% *γ*‐oryzanol, mixed with a HFD, resulted in significantly lower liver cholesterol and triacylglycerol levels than feeding rats only the HFD [18]. Based on these findings, we hypothesized that *γ*‐oryzanol might also affect the pathogenesis of MASH. We further considered whether it would be possible to investigate these effects using a MASH model in medaka. Consequently, we conducted experiments using *γ*‐oryzanol.

## Materials and methods

### Rearing conditions and feeding of medaka fish

According to the previous report [11, 12], 3‐month‐old Cab medaka (pure strain of *Oryzias latipes*) hatched and raised in the laboratory were used. The fish were kept in a still‐water tank inside a 24 °C incubator, using 1 liter of circulating water. The incubator maintained a 14‐h light/10‐h dark cycle, and aeration was provided within the tank. The water was changed every 2 days. Twenty‐one medaka were divided into three groups of seven and acclimated for 1 week on a standard diet (Hikari Labo 450; Kyorin). After 1 week, body weights were measured, and the following diets were administered for 12 weeks: (1) Control diet: 20 mg/fish (ND), (2) High‐fat diet (HFD32, Nihon CLEA): 20 mg/fish (HFD), (3) High‐fat diet + *γ*‐oryzanol (Wako Pure Chemical): 20 mg/fish (Ory). According to previous reports [11, 12, 19, 20], more than seven fish in a tank received a daily ration of 140 mg of the diet for that group, an amount that was completely consumed within 14 h. The protein–fat–carbohydrate (PFC) ratios and energy content per gram of each diet are shown in Table 1. The composition of the HFD and + Ory diets is shown in Table 2. The concentration of *γ*‐oryzanol added to the feed was based on previous studies that reported effects on the liver [18, 21]. All animal experiments were approved by the Institutional Animal Care and Use Committee (IACUC), Osaka Metropolitan University, and conducted in accordance with its animal experiment regulations. At Osaka Metropolitan University, mammals, birds, and reptiles are subject to review for animal experimentation, whereas fish are excluded from this review. Accordingly, no approval number is issued. This is because, in Japan, laws have been established that define mammals, birds, and reptiles as the subjects of animal experimentation, and our university conducts reviews in accordance with these regulations.

**Table 1:** PFC composition of feed and energy in this study.

Feed	Protein (%)	Fat (%)	Carbohydrate (%)	Energy (kcal/g)
ND	44.1	23.2	32.7	3.8
HFD	20.1	56.7	23.2	5.1
+Orz	19.6	56.9	23.5	5.1

**Table 2:** Composition of HFD and + Orz in this study.

Composition	HFD (g)	+Orz (g)
Milk casein	24.500	24.500
Safflower oil (high oleic type)	20.000	20.000
Powdered beef tallow	16.880	16.880
Maltodextrin	8.250	8.250
Lactose	6.928	6.928
Sugar powder	6.750	6.750
Crystalline cellulose	5.500	5.500
Egg white powder	5.000	5.000
Mineral mix (AIN‐93G‐MX)	5.000	5.000
Vitamin mix (AIN‐93VX)	1.400	1.400
L‐Cystine	0.430	0.430
Bitartrate choline	0.360	0.360
3‐Butylhydroquinone	0.002	0.002
*γ*‐Oryzanol	‐	0.500

### Measurement of body weight and liver weight / liver‐to‐body weight ratio

According to the previous report [11, 12], body weight was measured at the start of feeding and at Weeks 4, 8, and 12. During measurements and dissection, fish were anesthetized using FA100 (DS Pharma Animal Health). At Week 12, seven fish from each group were dissected. Abdominal and liver images were taken under a stereomicroscope (SZ‐40; Olympus), and liver weight was measured. The liver‐to‐body weight ratio was calculated to evaluate liver condition.

### Histological analysis

According to the previous reports [11, 12, 14, 22], histological analysis was performed. Livers collected at autopsy were embedded in Tissue‐Tek OCT compound (Sakura Finetek Japan, Tokyo, Japan) and frozen immediately, with at least 3 fish per group. Frozen tissue was thinly sliced (12 μm) using a rotary microtome (Cryostat HM525NX, PHC Holdings, Tokyo, Japan) and stored at −80 °C until staining. Before staining, the tissues were fixed in 4% paraformaldehyde‐phosphate buffer (Nacalai Tesque, Kyoto, Japan) for 10 min and stained with hematoxylin–eosin (HE) (Muto Pure Chemicals Co. Ltd., Tokyo, Japan), Oil Red O (Muto Pure Chemicals Co. Ltd., Tokyo, Japan), and Sirius Red (Muto Pure Chemicals Co. Ltd., Tokyo, Japan). Oil Red O staining was used to evaluate liver fat accumulation, and Sirius red staining was used to assess liver fibrosis [14]. After staining, the sections were sealed with an oil‐based sealant (Histomount, National Diagnostics, Atlanta, GA) for HE and Sirius Red, or a water‐soluble sealant (Fluoro‐Keeper, Nakalai Tesque, Kyoto, Japan) for Oil Red O. They were observed under an optical microscope (CX‐23; Olympus, Tokyo, Japan). For all stained tissues, images were captured with a digital camera (*α*‐7S; Sony, Tokyo, Japan) mounted on the microscope at 40x magnification at randomly selected positions. To evaluate the size of fat droplets in liver tissue, image analysis was performed using ImageJ (Fiji) [23]. The HE‐ or Oil Red O‐stained images, four per group, were converted to 8‐bit grayscale with a brightness threshold of 120–255.

### Fatty acid analysis by GC/MS

According to previous reports [11, 12], liver samples were collected from medaka fish at Week 12 of feeding for fatty acid analysis. Using a fatty acid methylation kit (Nacalai Tesque), fatty acids were extracted and methylated from the livers of four fish per group. The methylated fatty acids were then purified using a methylated fatty acid purification kit (Nacalai Tesque). Subsequently, the fatty acid content in the liver was measured using gas chromatography–mass spectrometry (GC/MS) (GCMS‐TQ8040 NX, Shimadzu Corporation). Nonadecanoic acid (C19 : 0) was used as the internal standard. The amount of each fatty acid per 1 mg of liver tissue was calculated based on the ratio of the peak area of the measured fatty acid to that of the internal standard. Fatty acid identification was performed using measurement data from alkane standards (RESTEK).

### Gene expression analysis of lipid metabolism via real‐time PCR

RNA was extracted from livers (about 30 mg) of each of 6 fish per group and immediately frozen after autopsy using an RNA extraction kit (RNeasy Mini Kit; Qiagen, Hilden, Germany). From the extracted total RNA, cDNA was synthesized using a cDNA synthesis kit (Transcriptor First Strand cDNA Synthesis Kit, Roche Diagnostics, Rotkreuz, Switzerland). Synthesized cDNA was mixed with primers and fluorescent dye (MyGo Green Mix Universal ROX, IT‐IS Life Science Ltd., Cork, Ireland) for two inflammatory cytokines and nine lipid metabolism‐related genes, respectively, and analyzed using a real‐time PCR system (qTower^3^, Analytik Jena, Jena, Germany). According to the manufacturer's manual, the run protocol was as follows: denaturation at 95 °C for 10 s, followed by 40 cycles of 95 °C for 10 s and 65 °C for 25 s. The internal standard gene was the 60S ribosomal RNA protein gene (*rpl7*) [24]. The four primer pairs were as follows: (1) Tumor Necrosis Factor‐alpha (*tnfa*) [10], (2) Interleukin 1‐beta (*il1b*) [25], (3) Peroxisome proliferator‐activated receptor alpha (*ppara*) [26], and (4) Sterol regulatory element‐binding transcription factor Element Binding Transcription Factor 1 (*srebf1c*) [26]. The primer sequences for each are shown in Table 3.

**Table 3:** Primer sequences for qPCR in this study.

Gene	Forward primer	Reverse primer
*srebf1*	GCCCTCCTGAACGATATTGA	AAACGTCGGTAGCTTCTCCA
*ppara*	GCTTTGTTCGTAGCCACCAT	GGACCTTCACGATGTTCTCC
*tnfa*	ATTGGAGTGAAAGGCCAGAA	AAACGTCGGTAGCTTCTCCA
*il1b*	GTCCAGCTGAACATGTCTAC	TTGTCTCCTTCTTGGTGGCA
*rpl7*	CGCCAGATCTTCAACGGTGTAT	AGGCTCAGCAATCCTCAGCAT

### Gut microbiota analysis

At Week 12 of feeding, intestinal contents were collected from medaka fish. Genomic DNA was extracted from the intestinal tract containing intestinal contents, collected and frozen at autopsy, using a DNA extraction kit (DNeasy PowerSoil Pro Kit, Qiagen). The intestinal contents of six or seven medaka per group were analyzed. The 16S rRNA gene region was amplified by PCR using the extracted DNA as a template. The amplified DNA fragments were purified and sequenced using a next‐generation sequencing platform (MiSeq, Illumina). Low‐quality terminals (quality score < 30) and adapters were removed from the obtained sequence data, and the data were denoised using the Deblur plug‐in of Qimme2. The silva‐138‐99‐classifier database was used to identify intestinal bacteria. The alpha diversity index (Shannon index) was calculated, and Bray–Curtis principal coordinate analysis (PCoA) was performed using the Bray–Curtis exponent as a measure of beta diversity. Moreover, we used linear discriminant analysis effect size (LEfSe) [27] to perform intergroup comparisons of the gut microbiota and to identify biomarkers.

### Statistical analysis

Statistical analyses were performed using the software Prism 9 (graphpad). To evaluate statistical significance between groups for variables such as body weight, liver‐to‐body weight ratio, histological analysis, fatty acid content, mRNA expression levels, and the abundance of bacterial ratio, one‐way analysis of variance (ANOVA) was conducted, and Tukey's method was used for multiple comparisons at *P* < 0.05 or 0.01. Alpha diversity in the assessment of the gut microbiota was analyzed using the Kruskal–Wallis test, while beta diversity was analyzed using permutational multivariate analysis of variance (PERMANOVA).

## Results

### Morphological changes and liver‐to‐body weight ratio

**Fig. 1:**
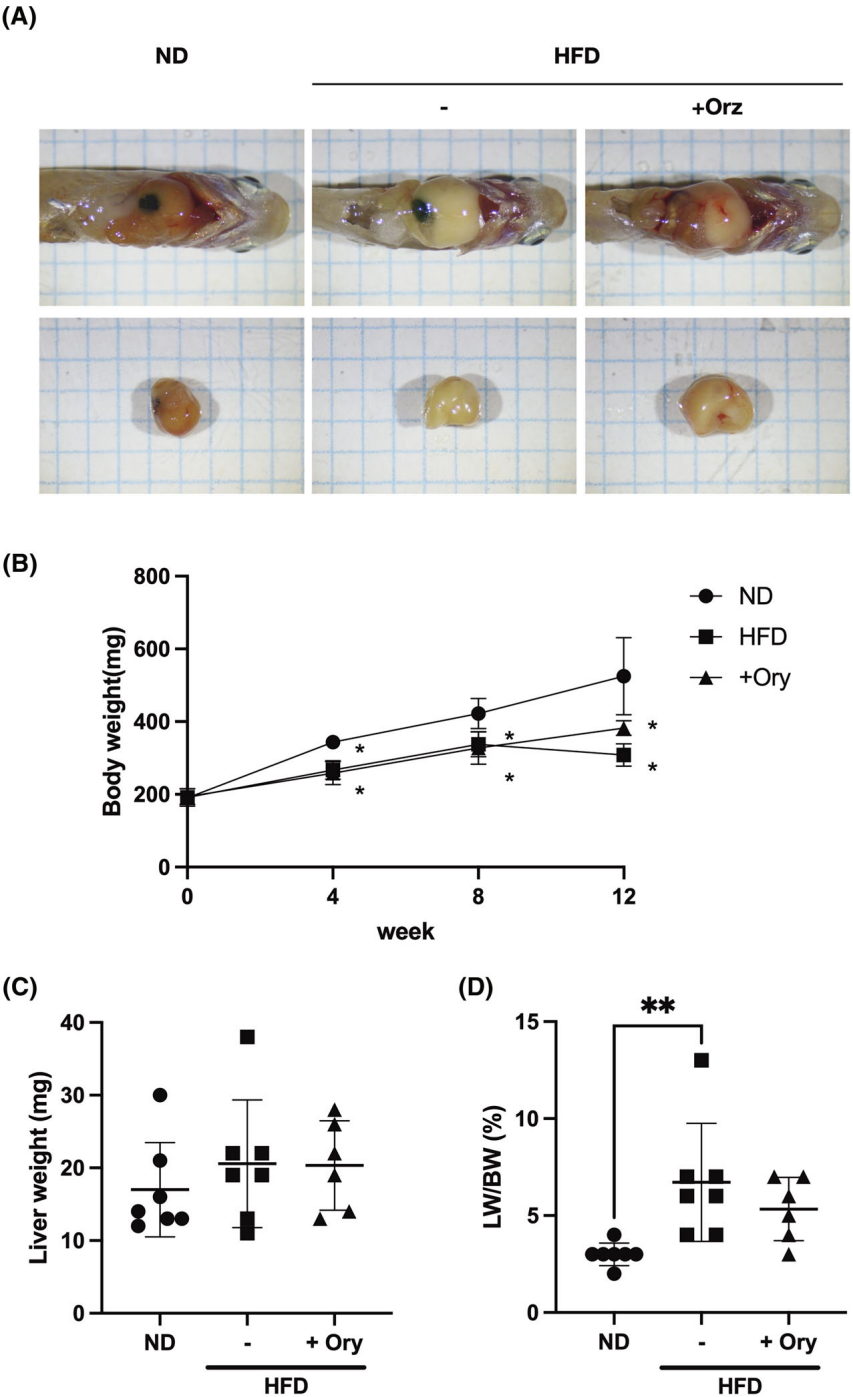
Morphological changes and body and liver weights in medaka after 12 weeks of feeding. (A) Top: During dissection, Bottom: Liver, photographed on 2 mm grid paper. (B) Changes in body weight during feeding. (C) Liver weight after 12 weeks of feeding. (D) Liver weight/body weight ratio after 12 weeks of feeding. Each group includes six or seven fish. Each data point in the graph represents the mean ± SD. Statistical analysis employed one‐way analysis of variance at the *P* < 0.05 significance level, with Tukey's test for multiple comparisons. *P < 0.05, **P < 0.01, ND: normal diet, HFD: high‐fat diet, +Orz: high‐fat diet mixed with *γ*‐oryzanol.

After 12 weeks of feeding with ND, HFD, and Ory diets, medaka fish were dissected and photographed to evaluate morphological changes. Compared with the ND group, the livers of the two HFD‐fed groups were noticeably whiter and enlarged. This change is believed to be due to fat accumulation in the liver resulting from a HFD. However, no apparent morphological differences were observed in the Ory groups compared with the HFD group (Fig. 1A). Body weight measurements were taken at the start of each diet, and at 4, 8, and 12 weeks after the start of feeding to evaluate changes in body weight. At 12 weeks after the start of feeding, the animals were sacrificed, and their livers were harvested and weighed. Body weight in the ND group was significantly increased at 12 weeks compared with that in the other 2 HFD‐fed groups (Fig. 1B). Meanwhile, no significant difference was observed; liver weight showed an increasing trend with HFD feeding (Fig. 1C). The liver‐to‐body‐weight ratio was then evaluated. The liver‐to‐body weight ratio was significantly increased in the HFD group compared with the ND group (Fig. 1D). However, no significant difference was observed in the Ory group compared with the HFD group (Fig. 1D).

### Histological changes in the liver

**Fig. 2:**
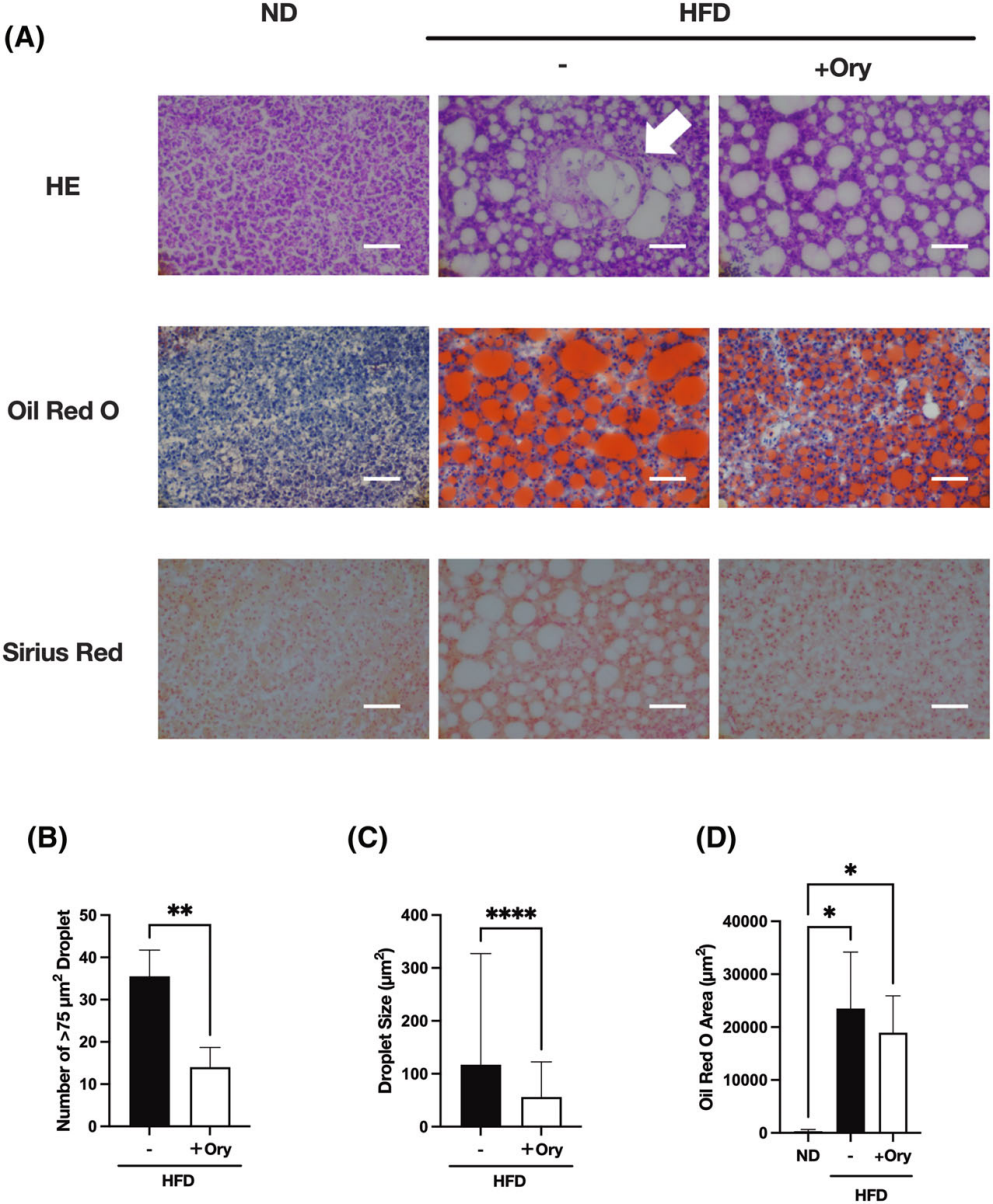
Histological changes in the liver 12 weeks after the start of feeding. (A) Three kinds of staining: hematoxylin–eosin (HE), Oil Red O, and Sirius red staining. Three fish per group were analyzed, and the typical images were shown. The white arrow shows a ballooning of hepatocytes in the liver. All images were captured at 40× magnification, with the scale bar indicating 50 μm. (B) Number of over 75 μm^2^‐fat droplets from HE images. (C) Fat droplet size from HE images. (D) Oil Red O Area from Oil Red O images. *P < 0.05, **P < 0.01, ****P < 0.0001, ND: normal diet, HFD: high‐fat diet, +Orz: high‐fat diet mixed with *γ*‐oryzanol. Each group consisted of three or four fish.

Twelve weeks after the start of feeding, medaka were dissected. Their livers were harvested for hematoxylin, eosin (HE) staining, Oil Red O staining, and Sirius red staining (Fig. 2A). HE staining revealed significantly more lipid droplets in the three HFD‐fed groups compared with the ND group, indicating lipid accumulation in the liver. Furthermore, ballooning of hepatocytes, a pathological feature of MASH, and inflammatory cell infiltration were observed in the liver section of the HFD group. However, no ballooning of hepatocytes was confirmed in the liver section of the Ory group (Fig. 2A upper). Larger fat droplets of the Ory group were decreasing compared with the HFD group (Fig. 2B,C). Oil Red O staining revealed, similarly to HE staining, a clear accumulation of fat in the two HFD‐administered groups compared with the ND group. Furthermore, areas with smaller fat droplets were observed in the Ory group compared with the HFD group (Fig. 2A,D, middle). Sirius red staining did not reveal obvious fibrosis in any of the HFD‐administered groups (Fig. 2A, lower panel). Furthermore, as with Oil Red O staining, smaller fat droplets were observed in the Ory group than in the HFD group (Fig. 2A, lower panel). Results from all three staining methods confirmed HFD‐induced fat accumulation in the liver. Additionally, administration of *γ*‐oryzanol suggested the potential to suppress liver fat accumulation.

### Analysis of liver fatty acid content by GC/MS

**Fig. 3:**
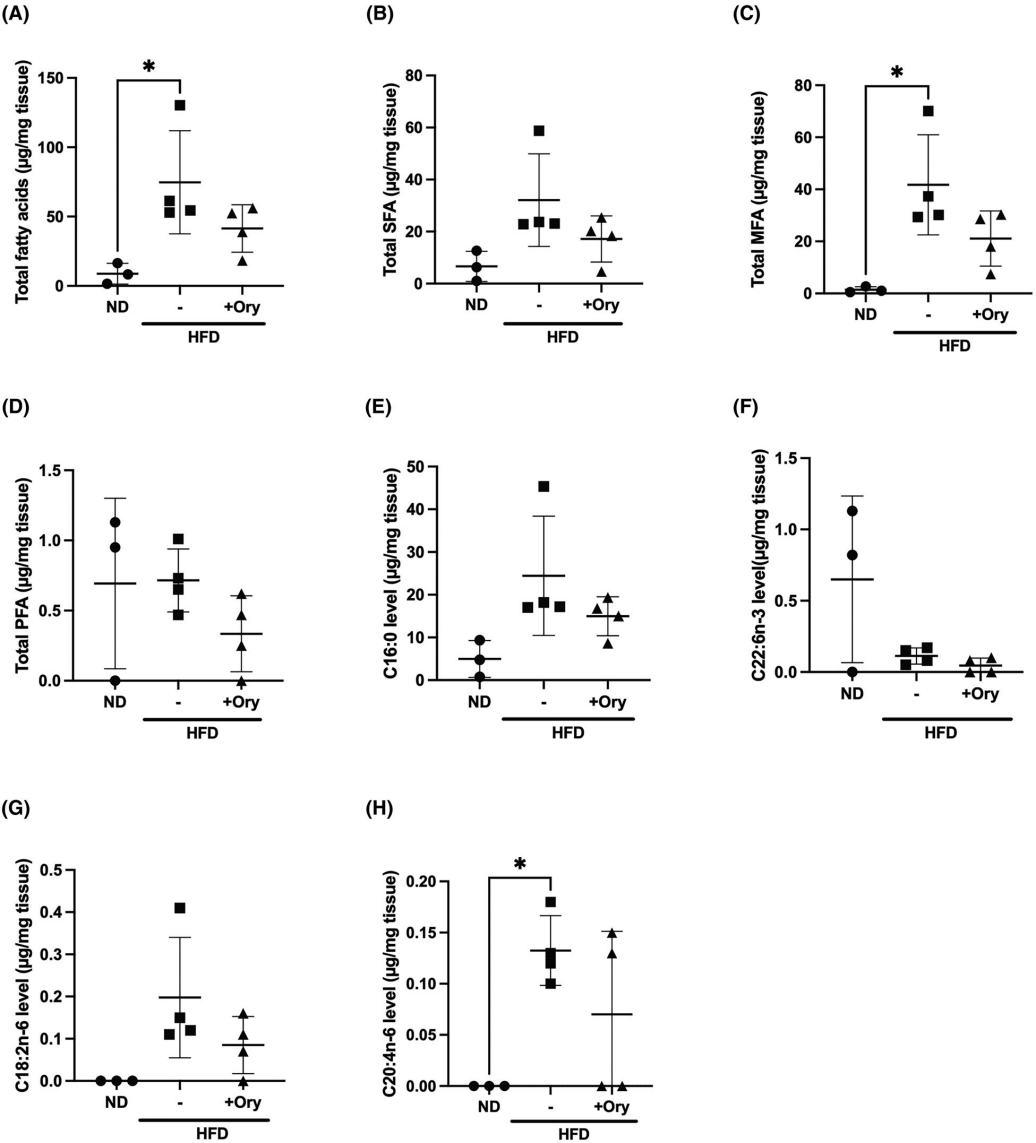
Fatty acid content per 1 mg of liver tissue in medaka from each group, 12 weeks after feeding initiation. (A) Total fatty acid content. (B) Saturated fatty acid content (SFA). (C) Monounsaturated fatty acid content (MFA). (D) Polyunsaturated fatty acid content (PFA) (E) Palmitic acid content. (F) Docosahexaenoic acid content. (G) Linoleic acid content. (H) Arachidonic acid content. Data points in the graph represent mean ± SD. Statistical analysis employed one‐way analysis of variance at a significance level of *P* < 0.05, with Tukey's test used for multiple comparisons. *P < 0.05. ND: normal diet, HFD: high‐fat diet, +Orz: high‐fat diet mixed with *γ*‐oryzanol. Each group consisted of three or four fish.

Liver tissue staining images suggested that HFD‐induced fat accumulation in the liver and *γ*‐oryzanol‐induced fat accumulation in the liver may be suppressed (Fig. 3). We therefore considered it necessary to measure and compare the lipid content and types of lipids in the liver. Using liver tissue from medaka after 12 weeks of feeding, we performed GC/MS analysis (Table 4). Total fatty acid content per mg liver was significantly increased in the HFD group compared with the ND group (Fig. 3A). Although no significant difference was observed in saturated fatty acid content (Fig. 3B) between the ND and HFD groups, an increasing trend was noted. Monounsaturated fatty acid content has a significant difference between the ND and HFD groups (Fig. 3C). These results confirm that HFD administration led to fat accumulation in the liver (Fig. 2A middle). In the Ory group, a decreasing trend was observed in total fatty acid content, saturated fatty acid content, monounsaturated fatty acid content, and polyunsaturated fatty acid content compared with the HFD group (Fig. 3A–D). These results, similar to Oil Red O staining and Sirius red staining, suggest that *γ*‐oryzanol may suppress fat accumulation in the liver. Among individual fatty acids, palmitic acid (C16 : 0), which is involved in the body's fatty acid synthesis pathway, showed an increasing trend in the HFD group compared with the ND group. The palmitic acid content also showed a decreasing trend in the Ory group (Fig. 3E). DHA (C22:6n‐3), abundant in fish oil, showed a decreasing trend in the HFD and Ory groups compared with the ND group. However, no clear difference was observed between the Ory group and the HFD group (Fig. 3F). EPA (C20 : 5n−3) was not detected in any of the groups. The essential fatty acids linoleic acid and arachidonic acid showed a remarkable increase in the HFD group compared with the ND group, and a tendency toward reduction in the Ory group compared with the HFD group (Fig. 3G,H).

**Table 4:** Amounts of fatty acids in the medaka liver of ND, HFD, and + Ory group.

Fatty acid	ND (μg/mg)	HFD (μg/mg)	+Orz (μg/mg)
Methyl myristate; 14:0	0.86 ± 0.52	3.18 ± 0.79	2.00 ± 0.53
Methyl pentadecanoate; 15:0	0.08 ± 0.05	0.40 ± 0.11	0.25 ± 0.05
Methyl palmitoleate;(Z) 16:1n‐7	0.21 ± 0.14	7.00 ± 1.87	3.45 ± 0.81
Methyl palmitate; 16:0	4.96 ± 2.48	24.44 ± 6.97	14.96 ± 2.28
Methyl cis‐10‐heptadecenoate;(Z) 17:1n‐7	0.00 ± 0.00	0.04 ± 0.03	0.01 ± 0.01
Methyl margarate; 17:0	0.02 ± 0.02	0.31 ± 0.10	0.16 ± 0.06
Methyl ganma‐linolenate;(Z) 18:3n‐6	0.00 ± 0.00	0.27 ± 0.06	0.10 ± 0.04
Methyl linoleate;(Z) 18:2n‐6	0.00 ± 0.00	0.20 ± 0.07	0.09 ± 0.03
Methyl cis‐7‐octadecenoate;(Z) 18:1n‐11	0.15 ± 0.07	4.70 ± 0.99	2.36 ± 0.58
Methyl linolenate;(Z) 18:3n‐3	0.00 ± 0.00	0.00 ± 0.00	0.00 ± 0.00
Methyl petroselate;(Z) 18:1n‐12	0.15 ± 0.07	4.70 ± 0.99	2.36 ± 0.58
Methyl oleate;(Z) 18:1n‐9	0.25 ± 0.12	6.53 ± 1.33	3.27 ± 0.77
Methyl linolelaidate;(E) 18:2n‐6	0.00 ± 0.00	0.20 ± 0.04	0.09 ± 0.03
Methyl cis‐vaccenate;(Z) 18:1n‐7	0.26 ± 0.13	6.75 ± 1.40	3.39 ± 0.80
Methyl elaidate;(E) 18:1n‐9	0.25 ± 0.12	6.53 ± 1.33	3.27 ± 0.77
Methyl cis‐12‐Octadecenoate;(Z) 18:1n‐6	0.14 ± 0.05	5.39 ± 2.01	2.95 ± 1.06
Methyl stearate; 18:0	0.72 ± 0.33	3.74 ± 0.98	2.52 ± 0.33
Methyl arachidonate;(Z) 20:4n‐6	0.00 ± 0.00	0.13 ± 0.02	0.07 ± 0.04
Methyl cis‐5,8,11,14,17‐Eicosapentaenoate;(Z) 20:5n‐3	0.00 ± 0.00	0.00 ± 0.00	0.00 ± 0.00
Methyl eicosa‐8,11,14‐trienoate; 20:3n‐6	0.00 ± 0.00	0.00 ± 0.00	0.00 ± 0.00
Methyl cis‐11,14‐Icosadienoate;(Z) 20:2n‐6	0.00 ± 0.00	0.00 ± 0.00	0.00 ± 0.00
Methyl cis‐11‐icosenoate;(Z) 20:1n‐9	0.00 ± 0.00	0.11 ± 0.04	0.03 ± 0.02
Methyl cis‐11,14,17‐Icosatrienoate;(Z) 20:3n‐3	0.00 ± 0.00	0.00 ± 0.00	0.00 ± 0.00
Methyl cis‐4,7,10,13,16,19‐Docosahexaenoate;(Z) 22:6n‐3	0.65 ± 0.34	0.11 ± 0.03	0.04 ± 0.03
Methyl cis‐7,10,13,16,19‐docosapentaenoate;(Z) 22:5n‐3	0.04 ± 0.04	0.00 ± 0.00	0.03 ± 0.03
Total saturated fatty acid	6.65 ± 3.35	32.08 ± 8.90	19.89 ± 3.20
Total monounsaturated fatty acid	1.41 ± 0.67	41.75 ± 9.62	21.10 ± 5.30
Total polyunsaturated fatty acid	0.69 ± 0.35	0.71 ± 0.11	0.34 ± 0.13
Total lipid	8.75 ± 4.33	74.74 ± 18.61	41.42 ± 8.55

### Gene expression analysis of lipid metabolism and inflammatory cytokines via real‐time PCR

**Fig. 4:**
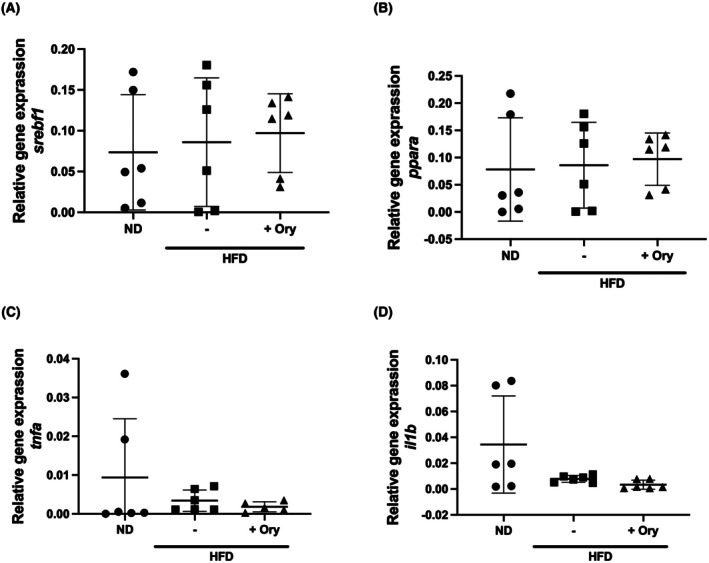
mRNA expression levels of lipid metabolism‐related genes and inflammatory cytokines in medaka liver 12 weeks after feeding initiation. (A) Sterol regulatory element‐binding protein 1 (*srebf1c*). (B) Peroxisome proliferator‐activated receptor *α* (ppara). (C) Tumor Necrosis Factor (*tnfa*). (D) Interleukin 1 *β* (*il1b*). Data points in the graph represent mean values ± SD. Statistical analysis was performed using one‐way analysis of variance (ANOVA) at a significance level of *P* < 0.05, with Tukey's *post hoc* test for multiple comparisons. ND: normal diet, HFD: high‐fat diet, +Orz: high‐fat diet mixed with *γ*‐oryzanol. Each group consisted of six fish.

To investigate the mechanism underlying the effects of *γ*‐oryzanol administration, we evaluated mRNA expression levels of genes related to lipid metabolism and inflammatory cytokines. No significant differences in mRNA expression levels of *srebf1c*, a gene regulating fatty acid synthesis, were observed across any group (Fig. 4A). Similarly, no significant differences in mRNA expression levels were observed for *ppara*, a gene regulating fatty acid *β*‐oxidation, across any group (Fig. 4B). On the contrary, HFD feeding groups indicate a reduction of mRNA expression levels of inflammatory cytokines TNF‐*α* (*tnfa*) and IL‐1*β* (*il1b*). However, no significant differences were found between the HFD and Orz groups (Fig. 4C,D), in contrast to previous mouse studies [28].

### Gut microbiota analysis

**Fig. 5:**
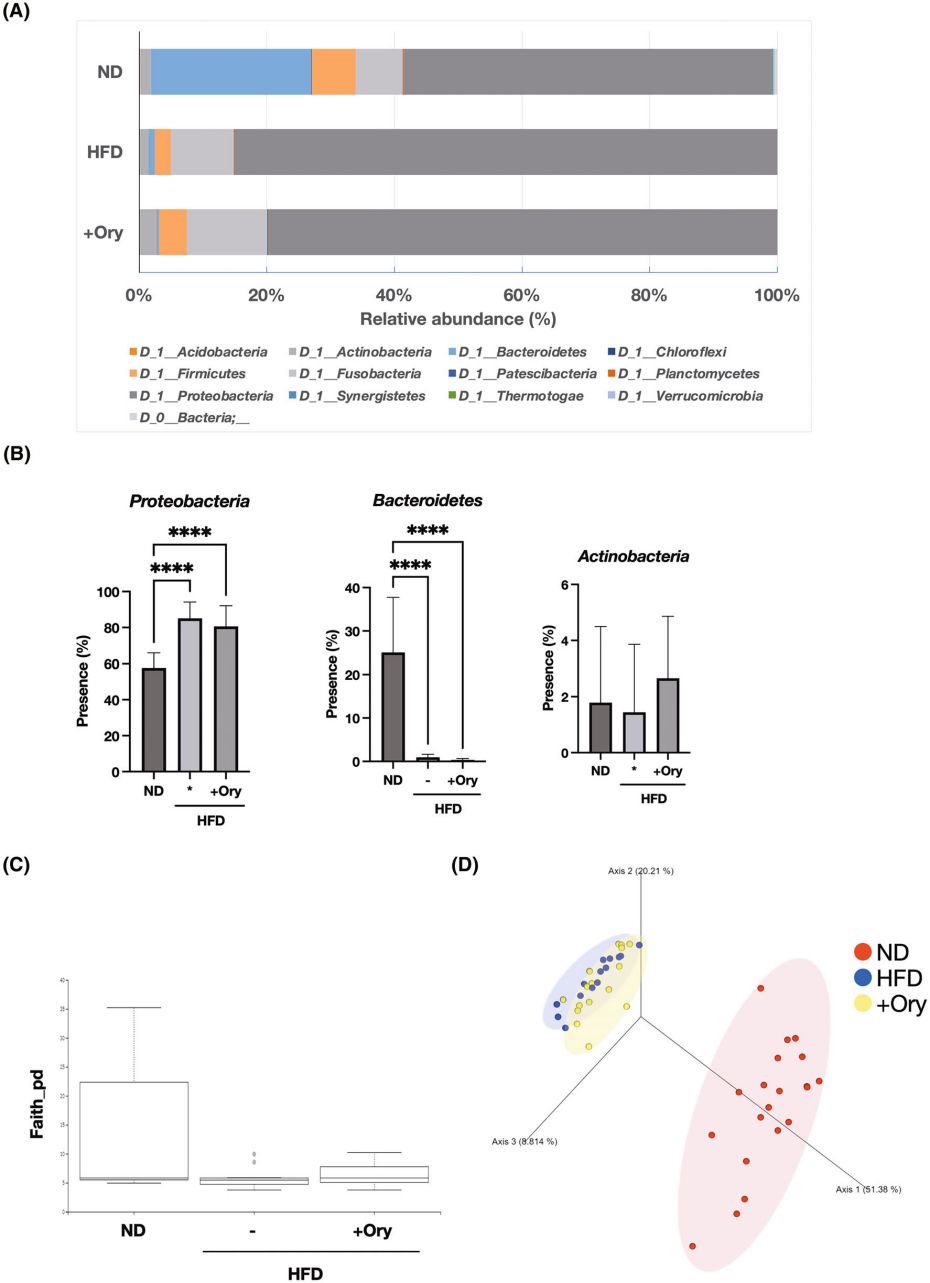
Analysis of medaka gut microbiota. (A) Composition ratio of bacteria (phylum level). The composition ratio was shown when the total of all bacterial species is set to 100%. (B) Relative abundance of several phyla. (C) *α*‐diversity. (D) *β*‐diversity. (B,C) Each data point in the graph represents the mean ± SD. Statistical analysis was performed using one‐way analysis of variance (ANOVA) at a significance level of *P* < 0.05, with Tukey's *post hoc* test for multiple comparisons. ****P < 0.0001. ND: normal diet, HFD: high‐fat diet, +Orz: high‐fat diet mixed with *γ*‐oryzanol. Each group consisted of six or seven fish.

**Fig. 6:**
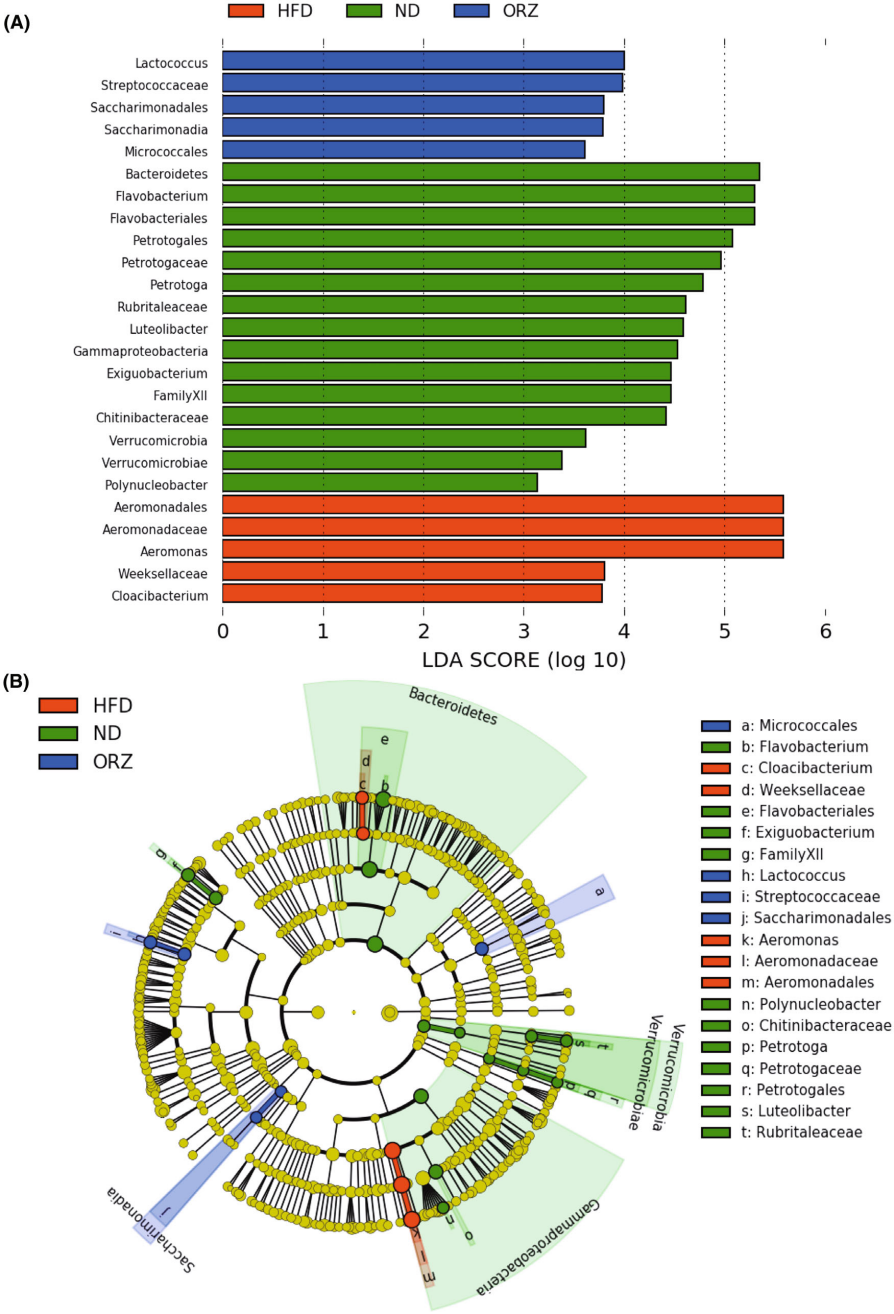
Linear discriminant analysis effect size (LEfSe) analysis of the gut microbiome. (A) The LDA scores, which serve as biomarkers. (B) The cladogram, which illustrates the similarity between groups based on the biomarkers. ND: normal diet, HFD: high‐fat diet, +Orz: high‐fat diet mixed with *γ*‐oryzanol.

Many reports have demonstrated that dysbiosis of the gut microbiota is associated with various human diseases and animal disease models. We also analyzed the gut microbiota in this study to assess changes in microbiota structure induced by *γ*‐oryzanol administration. Genomic DNA was extracted from medaka intestinal contents 12 weeks after the initiation of feeding, and 16S amplicon analysis was performed using a next‐generation sequencing platform. Based on the results, a study was conducted using Qiime2 to compare gut microbiota structure between groups. The graph showing the composition of bacteria at the phylum level revealed that the gut microbiota in the ND group was primarily composed of five types of bacteria (Phylum *Proteobacteria*, Phylum *Actinobacteria*, Phylum *Bacteroides*, Phylum *Fusobacteria*, and Phylum *Firmicutes*) (Fig. 5A). Compared with the ND group, it was revealed that the composition ratios of some of these five bacterial groups differed in the other two groups (Fig. 5A). Compared with the ND group, significant differences were observed in the *Proteobacteria* phylum and the *Bacteroides* phylum in the other two groups. In contrast, no significant differences were observed in the *Actinobacteria* phylum (Fig. 5B). Next, we calculated genus‐level abundance ratios and generated a graph of the 13 genera that accounted for the largest proportions. The graph showing the composition of bacteria at the genus level revealed that while multiple bacteria were present in roughly equal proportions in the ND group, there was a significant imbalance among bacteria in the other two groups (Supple Fig. 1). In the graph of the *α*‐diversity analysis, which indicates phylogenetic diversity within each group, it was found that diversity was significantly reduced in the HFD group compared with the ND group. Furthermore, when comparing the HFD group with the Ory group, although no significant difference was observed, the diversity of the gut microbiota in the Ory group tended to increase (Fig. 5C). In the three‐dimensional plot of the *β*‐diversity analysis showing diversity among groups, the ND group and the HFD group, the Ory group were widely separated; however, the Ory group was slightly closer to the ND group than the HFD group, suggesting that the composition of the gut microbiota was becoming more similar to that of the ND group (Fig. 5D). Furthermore, analysis of the gut microbiota at the genus level using LEfSe yielded LDA scores as biomarkers. In the HFD group, bacteria similar to the order *Aeromonas*, family *Aeromonadaceae*, and genus *Aeromonas* were identified. In contrast, in the Ory group, bacteria from the family *Streptococcaceae*, specifically the *genus Lactococcus, and from the orders Saccharimonadales, Saccharimonadia, and Micrococcales* (Fig. 6A). Based on these biomarkers, comparisons were made between groups (Fig. 6B).

## Discussion

In this study, a MASLD model using medaka fish was developed to evaluate the effects of food components on disease pathology and to compare with established MASH animal models. Specifically, the impact of 12 weeks of HFD feeding, with or without *γ*‐oryzanol, was assessed primarily through liver analysis.

The Ory group showed no significant morphological differences from the HFD group, perhaps due to similar diet composition and PFC balance. Previous studies in rats fed a high‐fat, high‐fructose diet supplemented with *γ*‐oryzanol showed reduced hepatic lipid droplets [18]. In this study, histological staining revealed smaller lipid droplets in the Ory group than in the HFD group, and GC/MS analysis showed a trend toward reduced total fatty acid content. *γ*‐Oryzanol has been reported to reduce ER stress in the hypothalamus caused by chronic animal fat intake, leading to decreased food consumption and improved glucose metabolism [17]. It also increased fecal lipid excretion in mice fed a HFD [16]. These effects may explain the reduced hepatic fat accumulation observed in this study. Since ER stress exacerbates MASH pathology [5], further investigation into *γ*‐oryzanol's impact on hepatic ER stress is warranted.

Regarding individual fatty acids, EPA was not detected, and DHA levels did not differ significantly between the Ory and HFD groups. These results may be attributed to the absence of DHA and EPA in the HFD itself, as well as the limited ability of fish and mammals to synthesize these fatty acids [29, 30]. Arachidonic acid is metabolized into pro‐inflammatory compounds, such as prostaglandin E2 [31]. Previous studies have reported that plasma levels of arachidonic acid and its metabolites are significantly elevated in MASH patients compared with non‐MASH patients [32]. Furthermore, it has been demonstrated that in rats fed a HFD, the arachidonic acid content in the liver increases significantly compared with that in controls [33]. In this study, arachidonic acid levels were lower in the Ory group than in the HFD group, despite similar dietary content, suggesting that *γ*‐oryzanol may reduce arachidonic acid accumulation. Future studies should evaluate related enzymes and gene expression to clarify the anti‐inflammatory mechanisms.

qPCR results show that mRNA expression of both TNF‐*α* and IL‐1*β* was apparently decreased in the two HFD‐fed groups compared with the ND group, without a significant difference (Fig. 4C,D). However, the Ory group was lower than the HFD group, suggesting a possible effect of gamma‐oryzanol, although there was no significant difference between the two groups. To our knowledge, no studies have investigated liver inflammatory cytokines using qPCR or ELISA in the high‐fat‐fed MASLD/MASH medaka model. Even in mice, some reports indicate that IL‐1*β* or TNF‐*α* levels are significantly elevated on HFDs, while others report substantial individual variation [34, 35, 36, 37]. Furthermore, it has been reported that the magnitude of the increase in pro‐inflammatory cytokines also varies by mouse strain [38]. Therefore, it will be necessary to consider strains other than the one used in this study for medaka as well. However, it has been reported that mice administered *γ*‐oryzanol concurrently with an HFD showed significantly reduced levels of inflammatory cytokines compared with mice on HFD alone [28]. Similar potential may be suggested in medaka from this study. As the mechanism underlying this remains unclear, further detailed investigation is necessary.

Regarding the effects of *γ*‐oryzanol on fatty acid metabolism, it has been reported that mRNA expression levels of genes encoding proteins involved in the fatty acid synthesis pathway decreased in human liver‐derived HepG2 cells following *γ*‐oryzanol administration [18]. However, precise data are currently scarce, and their effects remain unclear. In this study, GC/MS analysis showed a tendency toward reduced palmitate content in the Ory group. However, there were no significant differences in mRNA expression levels of SREBF‐1c and PPAR*α* in the liver between the HFD and Ory groups, leaving unclear whether *γ*‐oryzanol affects fatty acid metabolic pathways (Figs 3E and 4A,B).

One factor contributing to the progression of MASH is gut microbiota dysbiosis [6]. Conversely, *γ*‐oryzanol has been suggested to improve the gut microbiota potentially [39]. A study reported that consuming a fermented beverage rich in *γ*‐oryzanol for 1 month improved dysbiosis in the human gut microbiota compared with consuming a fermented beverage without *γ*‐oryzanol [17]. Furthermore, previous studies analyzing the gut microbiota of MASH patients have reported that, compared with non‐MASH patients, MASH patients exhibit a reduced proportion of the *Bacteroidetes* phylum and an increased proportion of the *Proteobacteria* phylum [40]. On the contrary, *γ*‐oryzanol is not water‐soluble, so 70% is not absorbed and is excreted in feces when administered orally; therefore, it is thought to be utilized by intestinal bacteria. Xia et al. [41] reported that in an inflammatory bowel disease (IBD) model rat induced by sodium dextran sulfate (DSS) administration, *γ*‐oryzanol administration increased the abundance of *Alloprevotella*, *Roseburia*, *Treponema*, *Muribaculaceae*, and *Ruminococcus*—all associated with short‐chain fatty acid (SCFA) synthesis—improving dysbiosis in the gut microbiota and alleviating disease symptoms. Analysis of gut microbiota in this study revealed that, based on the relative abundance at the phylum level, the disruption of the gut microbiota was slightly improved in the Ory group compared with the HFD group (Fig. 5A). According to previous studies, when the balance of the human gut microbiota is disrupted, a persistent increase in the *Proteobacteria* phylum is often observed [42]. In this study, compared with the ND group, the other two groups showed an increase in the *Proteobacteria* phylum (Fig. 5B). Furthermore, the *Bacteroides* phylum—considered a type of beneficial bacteria dominant in humans that metabolizes polysaccharides and oligosaccharides to supply nutrients and vitamins to the host and other gut bacteria [43]—was reduced in the other two groups compared with the ND group (Fig. 5B). This suggests that the gut microbiota is disrupted in both the HFD and Ory groups. On the contrary, although there was no significant difference, the phylum *Actinobacteria*, which is considered to play a crucial role in maintaining intestinal homeostasis [44], showed a tendency to increase in the Ory group (Fig. 5B). Furthermore, analysis of the composition at the genus level similarly revealed that the disruption of the gut microbiota was slightly improved in the Ory group compared with the HFD group (Fig. S1). Therefore, this suggests that the disruption of the gut microbiota was partially improved by feeding *γ*‐oryzanol. In the *α*‐diversity analysis, which indicates intragroup diversity, a significant decrease was observed in the HFD and Ory groups compared with the ND group; on the contrary, although there was no significant difference, a trend toward increased diversity was observed in the Ory group compared with the HFD group (Fig. 6A). Furthermore, in *β*‐diversity analysis, which indicates diversity between groups, the ND group, HFD group, and Ory group were widely separated; however, the distance between the ND group and the Ory group was slightly smaller than that between the ND group and the HFD group, with the former being closer to the ND group (Fig. 6B). These findings suggest that administering *γ*‐oryzanol to MASH model medaka slightly increases gut microbiota diversity. In the LEfSe analysis, completely different biomarkers were detected among the three groups (Fig. 6). According to previous studies, most species of the *Aeromonas* genus are opportunistic pathogens naturally distributed in diverse aquatic ecosystems; however, it has been reported that they proliferate under stressful conditions and cause infectious diseases in fish [45]. Furthermore, some lactic acid bacteria in the genus *Lactococcus* have beneficial effects on intestinal health [46]. In the HFD group of this study, the genus *Aeromonas* was identified as a biomarker, suggesting that a HFD was stressful for the medaka. On the contrary, in the Ory group, the genus *Lactococcus* was identified as a biomarker, suggesting that administration of *γ*‐oryzanol may have had a beneficial effect on intestinal health. Regarding *Sccharimonadales* and *Micrococcales*, there are no detailed previous studies, so a thorough discussion could not be conducted. Based on the above findings, it appears that administration of *γ*‐oryzanol to the MASH model medaka tends to improve somewhat dysbiosis—a disruption of the gut microbiota believed to be associated with the pathogenesis of MASH—and slightly increase gut microbiota diversity. In future studies, we plan to conduct a more detailed investigation by varying the duration and dosage of *γ*‐oryzanol administration and comparing the results.

This study suggests that while research using the medaka MASLD model is not currently common in the food sector, it could serve as an alternative to rodents if food components used in experiments are selected with consideration for feeding and husbandry characteristics. Regarding *γ*‐oryzanol, although only one concentration was added to the feed in this study, it is low‐toxic in rats [47]. Furthermore, since some *γ*‐oryzanol compounds are actually prescribed as treatments for dyslipidemia and as antidepressants, it is considered feasible to conduct experiments using higher feed concentrations. Increasing the dosage may reveal more pronounced improvement, necessitating further investigation into its impact on MASLD/MASH pathology and its underlying mechanisms.

## Conflict of interest

The authors declare no conflicts of interest.

## Author contributions

YI, HA, AY, and SK conceived and designed the project. YI, HA, HT, and AU acquired the data. YI, HA, JS, YK, AY, and SK analyzed and interpreted the data. YI, HA, and SK wrote the paper.

## Supporting information


**Fig. S1.** Analysis of the gut microbiota at the genus level.

## Data Availability

This manuscript contains supporting information.
