# Supplementary material for: Evaluating the effect of γ‐oryzanol on MASLD pathology using a medaka fish model
Source: FEBS Open Bio. 2026 Jun 30:10.1002/2211-5463.70301. Online ahead of print. doi: 10.1002/2211-5463.70301 (PMC13398859; doi:10.1002/2211-5463.70301)
Supplement: Supplementary file 1 — Fig. S1. Analysis of the gut microbiota at the genus level. [file FEB4-9999-0-s001.pdf]

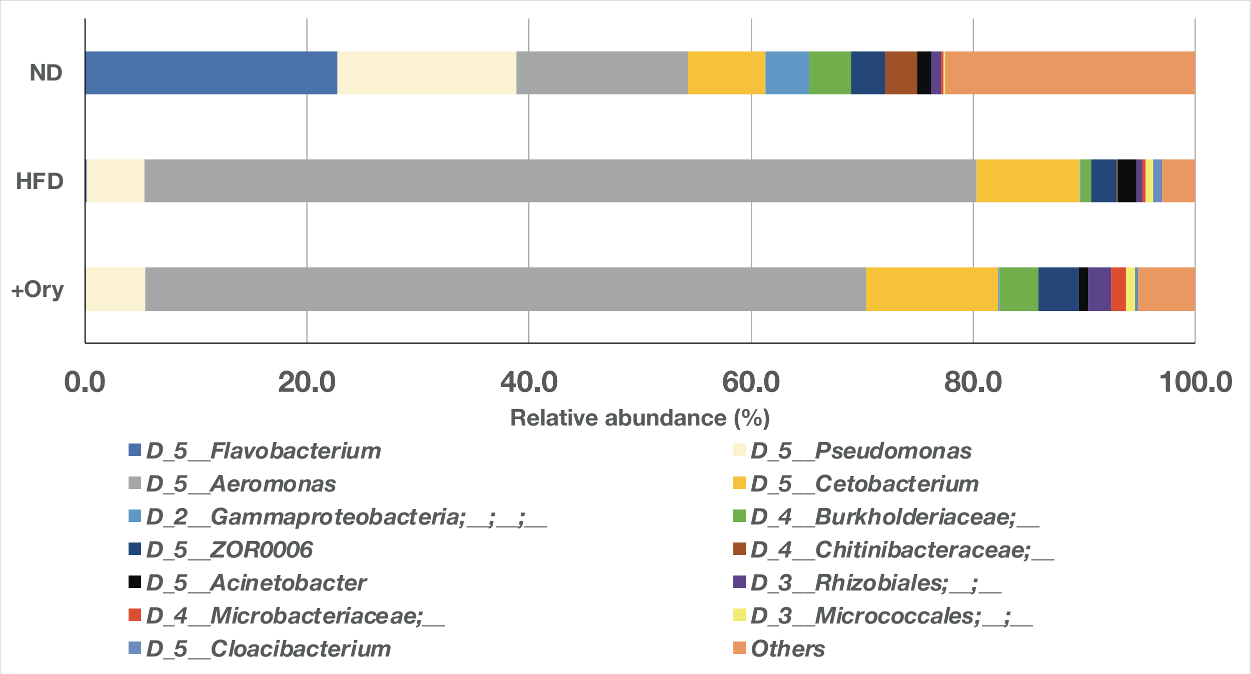

Supplemental Fig. 1 Analysis of the gut microbiota at the genus level

Composition ratio of bacteria (genus level). The composition ratio was shown when the total of all bacterial species is set to 100%. ND: normal diet, HFD: high-fat diet, +Orz: high-fat diet mixed with γ-oryzanol.
